# Analysis of Spatially Distributed Data in Internet of Things in the Environmental Context

**DOI:** 10.3390/s22051693

**Published:** 2022-02-22

**Authors:** Leonildo José de Melo de Azevedo, Júlio Cezar Estrella, Alexandre C. B. Delbem, Rodolfo Ipolito Meneguette, Stephan Reiff-Marganiec, Sidgley Camargo de Andrade

**Affiliations:** 1 Institute of Mathematics and Computer Science, University of São Paulo, Sao Paulo 13560-970, SP, Brazil; jcezar@icmc.usp.br (J.C.E.); acbd@icmc.usp.br (A.C.B.D.); meneguette@icmc.usp.br (R.I.M.); 2 School of Electronics, Computing and Maths, University of Derby, Kedleston Rd., Derby DE22 1GB, UK; S.Reiff-Marganiec@derby.ac.uk; 3 Computing Department, Federal University of Technology—Paraná, R. Cristo Rei, 19, Toledo 85902-490, PR, Brazil; sidgleyandrade@utfpr.edu.br

**Keywords:** Internet of Things, quality of data, data analyze, geostatistics, spatial statistics

## Abstract

The Internet of Things consists of “things” made up of small sensors and actuators capable of interacting with the environment. The combination of devices with sensor networks and Internet access enables the communication between the physical world and cyberspace, enabling the development of solutions to many real-world problems. However, most existing applications are dedicated to solving a specific problem using only private sensor networks, which limits the actual capacity of the Internet of Things. In addition, these applications are concerned with the quality of service offered by the sensor network or the correct analysis method that can lead to inaccurate or irrelevant conclusions, which can cause significant harm for decision makers. In this context, we propose two systematic methods to analyze spatially distributed data Internet of Things. We show with the results that geostatistics and spatial statistics are more appropriate than classical statistics to do this analysis.

## 1. Introduction

Nowadays, it is possible to easily access services and data through the Internet from any place and at any moment. It can be observed from recent decades that computational resources are becoming increasingly accessible and more powerful. Furthermore, the number of devices connected at the Internet has increased exponentially increase and is projected to amount to 75.44 billion worldwide by 2025 (https://www.statista.com/statistics/471264/iot-number-of-connected-devices-worldwide/ (23 November 2020)). According to Cisco Annual Internet Report (2018–2023) (https://www.cisco.com/c/en/us/solutions/collateral/executive-perspectives/annual-internet-report/white-paper-c11-741490.html), the number of devices connected to Internet Protocol (IP) networks will be more than three times the global population by 2023. However, these numbers only refer to devices such as computers, smartphones, and tablets; if considered other devices such as sensors, this number would be double easily. With many connections, devices communicating with humans and other devices have enabled the development of a paradigm called the Internet of Things (IoT) [1].

IoT involves anything with network access, for instance, sensors to advise on localized fertilizer amounts or targeted pesticide use, self-monitoring health systems, air quality, and traffic routing [2,3]. These sensors have the ability to transfer data over a network with or without requiring humans, and these data can be provided in many forms, such as streaming and discrete data, images, and social media, among others. The combination of sensors network with the Internet enables the communication between the virtual and the real world, allowing the decision-making without human intervention.

According to economic analysis from Cisco, “IoT will generate $8 trillion worldwide in Value at Stake over the next decade. This will come from five primary drivers: innovation and revenue ($2.1 trillion); asset utilization ($2.1 trillion); supply chain and logistics ($1.9 trillion); employee productivity improvements ($1.2 trillion); and enhanced customer and citizen experience ($700 billion)” (https://newsroom.cisco.com/press-release-content?articleId=1621819). By not considering many factors that involve quality of service or even a correct data analysis, it can probably cause financial losses to organizations. Some real cases can be cited, such as the following: (1) Gartner has an annual cost because of poor data in 2014 on average of $13.3 million dollars [4]; (2) The US Postal Service has finance losses over $1.5 billion due to mail with wrong data [5]. The US economy has finance losses of over $3 trillion a year [6].

The problem of data quality becomes complex and controversial with technology evolution. With significant financial losses caused by weak data, these problems have become the focus of much research from many perspectives. However, most of these works are dedicated to solving a specific problem in a particular environment. With close flow, it is difficult to consider the real capacity of IoT, since there is no sharing of information. Furthermore, another problem is the accuracy of data quality in decision-making.

The data quality and data accuracy are also related to the data analysis [7,8,9]; i.e., an incorrect data visualization or wrong method analysis could lead to misinterpretations or wrong decision making, even if the data are collected correctly. In this context, this article puts forward a systematic approach to support the data analysis by considering the sensor spatiality factor and geographic aspects. To validate this approach, we applied the methods on an extensive real-world database from the United States Environmental Protection Agency (US EPA), specifically involving air quality data; we describe the dataset in Section 4. The main contributions of the paper are as follows:

- A data analysis approach for outdoor sensors based on geostatistic data: a non-classic statistical approach to IoT data analysis, which it is not used on the majority works, due to the data limitation, the  scenario space of the analysis, and the fact that the data are not from the real world;
- A data analysis approach for outdoor sensors based on spatial statistics: like the above-mentioned approach, however, here we analyze data in a discrete space (delimited by a boundary), and in geostatistic data, it considers a continuous geographic area;
- A structuring of several methods from geostatistics and spatial statistics aggregated with a multicriteria analysis to compose a systematic data analysis on outdoor sensors: this is our main contribution, where we structured an outdoor sensors’ data analysis approach considering the geographic data dispersion and conflicted indicators;
- An assessment of the proposed method and comparing other works that apply classical statistics.

The rest of the paper is organized as follows. In Section 2, is works related to IoT data quality and data analysis. Section 3 introduces essential concepts to the method. The proposed method analysis is described in Section 4. A case study to apply the methods is presented in Section 5. The application and comparison with the existing techniques are described in Section 6. Finally, Section 7 discusses the outcomes and recommendations for further work.

## 2. Related Works

The Internet of Things is a highly scalable environment in which the data generated are tremendous. Thus, the quality of information is becoming an issue of great interest in both the academic and the industrial worlds. In this section, we discuss some of the works related to data quality in IoT. Moreover, we also discuss the practices related to the application domain of this paper and the related works to the methods that we proposed as a solution to make the best data analysis with spatially distributed data.

### 2.1. Data Quality in Internet of Things

There are many works in the literature that address quality of service and data manipulation in IoT. For instance, some works apply a publish–subscribe methodology to simplify the integration between sensors and the cloud [10,11,12]. However, these solutions do not assess the accuracy of the data or the analysis.

Other works try to apply particular solutions, such as a model-driven framework, to data quality management [13], and a Blockchain-based approach was attempted in [14]. These solutions aim to improve IoT data quality and false data detection. On the other hand, the solutions are applied in specific architectures and do not present a robust analysis of the generated data.

There are some authors who propose solutions on ontology-based [15,16], where they had a focus on identifying missing data or using the quality of information as an indicator of IoT trust [15]. Although these solutions even present a math solution model, they do not present an assessment or application evaluation of the real-world environment or even real data.

In [17], the authors propose an attractive solution for data cleaning by an incorrect data detection method based on an improved local outlier factor. Although the proposed method was used to detect inaccurate data from offline data, the solution achieved excellent performance to identify poor data. However, this solution identifies the incorrect data only from the collection point and does not consider the visualization or analysis method.

Another work with a similar proposal is [18], where the authors developed a data quality analysis and cleaning strategy for wireless sensor networks. For this, the authors studied the impact of the relationship between different indicators on the quality assessment during data cleaning. Although the authors performed some simulations, they did not evaluate the solution in a real-world environment; moreover, just like the previous work, they considered only the data from the sensor’s point.

There are also several other works related to the quality of data originating from the sensors [19,20,21]. In [19], the authors designed a prototypical implementation of a distributed IoT middleware layer to manage heterogeneous data sources. In [20], the authors propose an altruistic approach to data quality assessment for sensor data. Furthermore, in [21], the authors present a framework to evaluate and control data quality aspects when dealing with social and sensor data. However, all of these works address only the data quality in the collection point and specific scenarios; our proposal aims to show how to visualize and build a correct analysis with IoT spatially distributed data.

The authors of [7], specifically disucss the state of the art of the data quality of the Internet of Things. According to [7], the data generated in global scale deployment are tremendous, and there are many open challenges related to data quality. The authors also presented a detailed survey about quality features and the significance of a robust and accurate data analysis. In this paper, we apply geostatistics and spatial statistics to make a precise data analysis in IoT on the environmental context.

### 2.2. Environment and Pollution Context in IoT

To evaluate our proposal, we applied the methods on an extensive real-world IoT database from the United States Environmental Protection Agency (USEPA), which we described in Section 4. Notably, the environment subject is also a relevant research topic. For this reason, we also researched in the literature on how the data are analyzed in this field.

Exciting work in this field analyzed the impact of COVID-19 on people’s lives and the natural environment [22]. For this purposed, the authors investigate the spatial and temporal characteristics of the Air Quality Index (AQI) before and during the pandemic in mainland China. The authors present several analyses with respect to this theme; however, all of them apply classical statistical analysis. In this paper, we show that IoT spatially distributed data request a different interpretation.

There also other works that utilized the USEPA dataset to analyze the environmental context [23,24]. In [23], the authors conducted a comparative study of AQI based on factor analysis and USEPA methods for an urban environment. Furthermore, in [23], the authors did not use the USEPA but used the same recommended method for health risk assessment in a similar dataset in China. In both works, the authors used traditional statistics to analyze specific points, which could not show the real context of the region.

In the same field, there is a project being conducted at the Alan Turin Institute called London Air Quality (https://www.turing.ac.uk/research/research-projects/london-air-quality). This project utilizes city-wide air quality sensors to develop solutions to understand and improve air quality over London. This group’s research has achieved impressive results by applying machine learning algorithms and proposing data science platforms [25,26,27,28,29,30]. In this paper, we propose a different solution by spatial autocorrelation analysis, focusing on data analysis and data visualization.

### 2.3. Spatial Autocorrelation

Spatial autocorrelation is an association indicator from Geographic Information Science (GIScience) [31,32]; we discuss this in Section III. This theme has been subject of many studies [33]. In [34], the authors discuss the big spatiotemporal data analytics as a research and innovation frontier, and one of the fields that is considered promising is the IoT.

There are in the literature some authors who propose applying geostatistics in the IoT environment in many different ways [35,36,37]. However, these works do not demonstrate the application method with concrete results, and they also do not propose a systematic way to apply the techniques—some of them only discuss the potential.

In a recent study [38], the authors investigated rainfall-related tweets to determine the areal units that optimize spatial autocorrelation patterns through the combined use of indicators of global spatial autocorrelation and the variance of local spatial autocorrelation. In our study, we propose using the same technique to scale the ideal areal units to analyze the data.

In this paper, we propose a systematic approach to support the data analysis and the decision makers by considering the sensor spatiality factor and geographic aspects. For this purpose, we applied methods from the spatial statistics and geostatistic fields.

### 2.4. Proposal Highlight

To highlight our contribution, we present in Table 1 the main features of the related works, with the following columns:

- **Related work:** reference to the related work addressed;
- **Environment:** the experimental environment, either *Real world* (e.g., a prototype) or *Simulator* (i.e., a simulated experiments in a fictitious environment);
- **Spatial:** whether the approach considers the spatial dispersion in the analysis;
- **QoD:** whether the approach considers the QoD attributes in the data analysis;
- **Multi-criteria analysis:** whether the approach treats the problem as a multi-objective problem and/or considers any conflicting objectives.

By analyzing Table 1, we can observe that our proposal focuses on accurate analysis. For this purpose, we use only real-world data to validate our method, geostatistics and spatial statistics to consider the spatial data dispersion, and a multicriteria analysis to resolve the conflicting objectives. We present the results below.

## 3. Geographic Information Science

Spatial statistics and geostatistics are methods from the Geographic Information Science (GIScience) field that encompass a wide array of disciplines, such as geography, cartography, geodesy, statistics, and computer science. GIScience considers the nature of geographic information to develop theories and methods for understanding geographic processes, relationships, and patterns at different geographical scales [31,32]. GIScience also includes social disciplines that address issues and impacts on society.

### 3.1. Spatial Data Analysis

In the GIScience field, the spatial data analysis is consider a central topic. It deals with “a collection of techniques and models that explicitly use the spatial referencing associated with each data value or object that is specified within the system under study” [39]. These methods are crucial to assess spatial relationships and assumptions in spatially distributed data.

There are two fundamental concepts in spatial data analysis: (1) spatial autocorrelation, which refers to the degree of dependence from similar objects near to others, and (2) spatial heterogeneity, which is related to structure of these objects [40]. Analyzing these concepts makes it possible to answer questions such as “how much does the economics of one neighborhood influence another?” and we also hope to answer the questions “what is the correct areal unit to analyze a set of sensors?” and “How can spatially distributed data be analyzed?”

### 3.2. Spatial Autocorrelation

The geography scale, aggregation, and detail level are essential to construct an appropriate representation of the world, i.e., according to the process of handling the aggregation of delimited the unit spaces, the data could show different values and interpretations [40]. In this context, different measures from the real world can covariate, and understanding the spatial correlation essence could help to understand the analyzed phenomena better.

Spatial autocorrelation is directly related with the first law of geography or Tobler’s law, which says “everything is related to everything else, but near things are more related than distant things” [41]. This law is a fundamental premise for spatial statistics, and could also be interpreted as a definition for the **positive spatial autocorrelation**. The opposite of the law implies a **negative spatial autocorrelation** when places close to each other have high spatial heterogeneity.

The interrelation between the features of a location is an essential aspect of the geography data, which is crucial for real-world comprehension [42]. However, this interrelation is a challenge for classic statistics due to the majority method to consider the independence of the observations without spatial correlation.

## 4. Methods

To analyze spatially distributed data in IoT, we propose the use of two methods from the GIScience field. The first one (statistical spatial) is a framework proposed by [38] based on Moran’s index [43], and the second one (geostatistic) is an interpolation method for a correct data visualization [44]. Table 2 describes the main variables used in this work.

### 4.1. A Framework to Definition of the Spatial Granularity

To measure the spatial autocorrelation level, it is possible to use an index that may vary between 1 and −1: 1 for the high positive spatial autocorrelation, −1 for high negative spatial autocorrelation, and 0 for the absence of spatial autocorrelation [45].

There are two types of indexes for this association: a global and other local. The global coefficient correlation measures the overall spatial autocorrelation of the data set, with only one index value. On the other hand, the local indicator of spatial autocorrelation (LISA) measures different levels of spatial relationships; it depends on the scale defined, such as district, county, state, country, etc.

The most common global and local indexes are calculated by Moran’s *I*. The global Moran’s *I* is the result of the Equation (1) [46].

(1) $$I=\frac{n}{\sum_{i}^{n}\sum_{j}^{n}w_{ij}}\cdot \frac{\sum_{i}^{n}\sum_{j}^{n}w_{ij}\left(y_{i}-\bar{y}\right)\left(y_{j}-\bar{y}\right)}{\sum_{i}^{n}{\left(y_{i}-\bar{y}\right)}^{2}}$$

where

$w_{ij}$, is the matrix unit weight, $w_{ij}=1$ if *i* and *j* are neighbors, and $w_{ij}=0$ otherwise;
$y_{i}$ and $\bar{y}$ represent the value and the mean of interest on location *i*;
*n* is the total observations; and, *I* is the Moran’s index, a metric used to test the hypothesis about spatial autocorrelation.

The Moran’s *I* aims to test the spatial independence (null hypothesis). In this context, the null hypothesis is true if its value is zero. Positive values, between 0 and 1, point to a positive autocorrelation, and negative values, between 0 and −1, indicate negative autocorrelation.

This local indicator utilization together with the global index improves knowledge about the process from which the spatial dependence originates. The LISA makes a specific value for each object, which can identify clusters, outliers, and the existence of more than one spatial pattern.

According to [46], a LISA should adhere to two objectives: (1) to allow the identification of significant spatial associate patterns and (2) to be a decomposition from the global spatial association index. Equation (2) show Moran’s LISA calculation.

(2) $$I_{i}=\frac{\left(y_{i}-\bar{y}\right)\sum_{j=1}^{n}w_{ij}\left(y_{j}-\bar{y}\right)}{\frac{\sum_{i=1}^{n}{\left(y_{i}-\bar{y}\right)}^{2}}{n}}$$

where

$w_{ij}$, is the matrix unit weight, $w_{ij}=1$ if *i* and *j* are neighbors, and $w_{ij}=0$ otherwise;
$y_{i}$ and $\bar{y}$ represent the value and the mean of interest on location *i*;
*n* is the total observations; and, $I_{i}$ is the Moran’s LISA for each map unit.

In Equation (2), an $I_{i}>0$ means that *i* has values very similar to its neighbors (positive spatial autocorrelation), and $I_{i}<0$ means that *i* has different values from the neighbors (negative spatial autocorrelation). Furthermore, analogously to the global indicators, the Moran’s LISA should be evaluated by the pseudo-significance test.

As demonstrated in [38], the determination of an optimal areal unit for spatial analysis is a complex task owing to the Modifiable Areal Unit Problem (MAUP) effects, differences in the fields of application, and uncertainties and conflicts arising from the different potential spatial indicators to be used. For this reason, it is necessary to select the candidate solution (optimal areal unit) by a Pareto ranking [47].

To apply Pareto ranking in this framework [38], in order to model a solution, let *X* be a set of any areal units with different levels of data aggregation. Each spatial granularity of aggregation x∈X is characterized by different criteria that will be optimized by a set of objective functions; in this case, the global and local indexes. A vector containing *m* objective functions $\phi_{m}$ can be represented by

(3) $$\Phi \left(x\right)=\left[\phi_{1}\left(x\right),\phi_{2}\left(x\right),\cdots ,\phi_{m}\left(x\right)\right]\in R^{m}$$

A Pareto-optimal solution only contains areal units that are not Pareto-dominated by any other areal unit [38]. In general terms, an areal unit $x_{i}\in X$ dominates another $x_{j}\in X$ when it has satisfied the following two constraints:

(i) $\forall \phi \in \Phi :\phi \left(x_{i}\right)⪯\phi \left(x_{j}\right)$, and
(ii) $\exists \phi \in \Phi :\phi \left(x_{i}\right)≺\phi \left(x_{j}\right)$

where ≺ and ⪯ correspond to the ‘general better’ and ‘better or equal’ relations, depending on whether the objective function refers to maximization or minimization. It is possible to obtain more than one Pareto Frontier according to the ranking or even two or more solutions in the Pareto-optimal areal units; in this case, additional human expertise is required for the selection of a proper areal unit.

In Algorithm 1, we present a systematic way to use this framework. First, we provide the input data (line 1); in this paper, we use a pollution data set described in Section 5. The first step of the method is to model the candidate’s areal unit solution, and here it defines the size of the areal unit to make the data aggregation (line 3). In the second step (line 4), it assesses the candidate’s areal unit by the defined criteria; in this case, they are the global and local autocorrelation index (Global Moran’s *I* and the coefficient of variation of Local Moran’s *I*, respectively). The last step is to select an “optimal” areal unit from the non-dominated Pareto frontier (line 5).
**Algorithm 1** Multicriteria for the selection of an optimal areal unit1:Input data: pollution data at an individual level (the pollution data in our application)2:**for** each areal unit on set of criteria, **do**3:    Modeling of candidate areal unit4:    Evaluation of an candidate areal unit (MCDA)5:    Selection of the optimal areal unit (non-dominated solution)6:**end for**7:**return** Optimal areal unit

### 4.2. Data Interpolation

**Figure 1:**
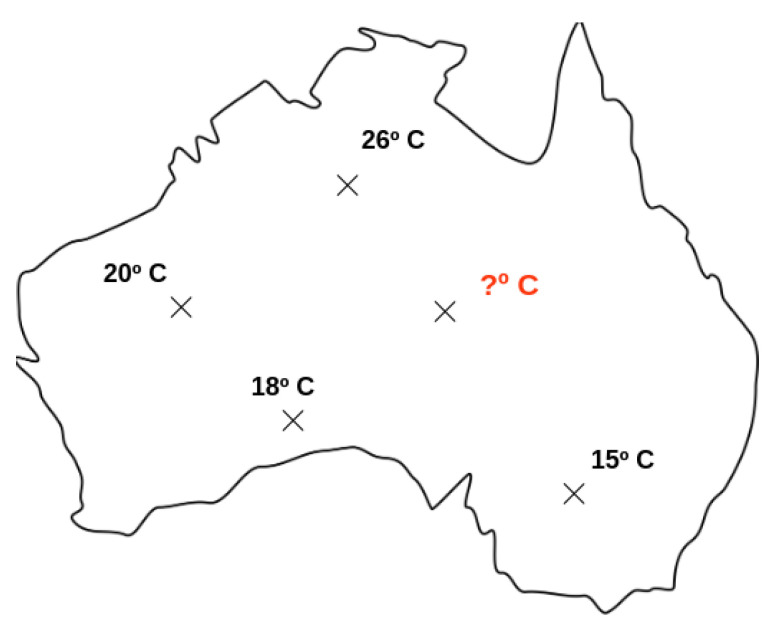
Example of the need to estimate a value that does not have spatial information available.

For a coherent data visualization and correct data measure, we apply a data interpolation method, namely Kriging [44]. This technique is a regression method from geostatistic to data interpolation, i.e., to estimate values in unknown data points. In Figure 1, we show an example situation, where we would like to know the temperature from a local that does not have spatial information available.

There are many other data interpolation techniques in the GIScience field [42]. However, the Kriging method allows for incorporating three factors to improve the estimation accuracy: (1) local fluctuation, which makes it possible to analyze the spatial autocorrelation during the data interpolation; (2) noise, which makes it possible to identify random changes space independent, i.e., detect errors in the collected data; and (3) incorporating general trends as an auxiliary variable, e.g., using a model with similar behavior to help in the estimation. More details about any of those factors can be found in [42].

Kriging’s technique measures the surrounding values to derive a prediction for a location with unknown data. The Kriging interpolation formula is formed as a weighted sum of the data, as described in Equation (4).

(4) $$\hat{Z}\left(S_{0}\right)=\sum_{i=1}^{N}\lambda_{i}Z\left(S_{i}\right)$$

where

$Z(S_{i})$ is a known value at the location *i*,
$\lambda_{i}$ is an unknown weight for the measured value at the location *i*,
$S_{0}$ is the location with data unknown to the prediction, and
*N* is the number of measured values.

In the Kriging method, the $\lambda_{i}$ is dependent on a fitted model to the value locations, the spatial relationship among the known values that surround the prediction location, and the distance from the known points to the prediction location. Therefore, it is necessary to create the variograms and covariance functions to estimate the statistical dependence to make a fitted model to the measured points. Details about the fitted model features, as well the variograms and covariance functions, can be found in [42].

**Figure 2:**
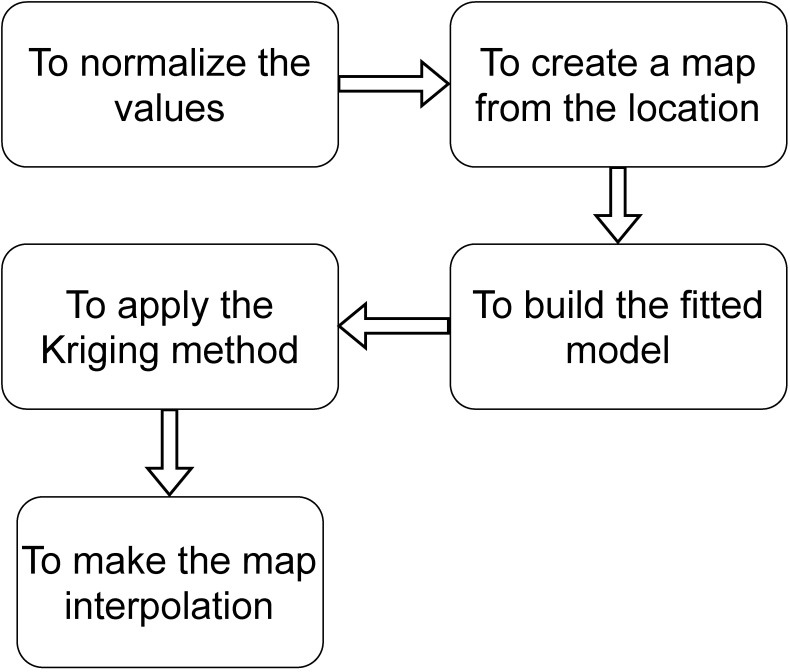
A systematic way that we use to apply the Kriging interpolation on the IoT context.

We show in Figure 2 the systematic way that apply the Kriging interpolation in the IoT context. First, we normalize the input data and build a shapefile from the local area; the map is only for visualization. The second step is to model the variogram (i.e., to construct the fitted model) and then apply the Kriging method. The last step is to make the map interpolation.

To normalize the data values, we use the *bestNormalize* (https://cran.r-project.org/web/packages/bestNormalize/index.html) package from the R language. Furthermore, we developed all of the systematic methods in R, which are available in https://github.com/Leonild/SpatialDataAnalysis.

## 5. Case Study

In recent years, high levels of pollution in specific dry periods of the year have forced authorities to rethink the organizational strategy of cities and propose drastic changes in urban centers. According to the World Health Organization (WHO) (https://www.who.int/), half of the world’s population lives in urban centers, and the estimate for 2050 is that 70% of the population will be urban [48]. This means that urban development will have a direct impact on human health.

Human health is affected by several correlated factors, factors that go beyond the power of health agencies. These include residences, sanitation, transportation, the energy system, and parks with green spaces, in addition to decent jobs, education, and healthy food [49].

With population growth, by 2050, it is estimated that 2.5 billion people will inhabit cities in addition to those who already inhabit them. This presents a unique opportunity to plan cities that protect and promote public health through well-structured organization. In this context, pollution has drawn a great deal of attention, causing irreversible damage to the planet, as well as global warming, respiratory diseases, and extinction of microbiomes, among others [50,51].

**Figure 3:**
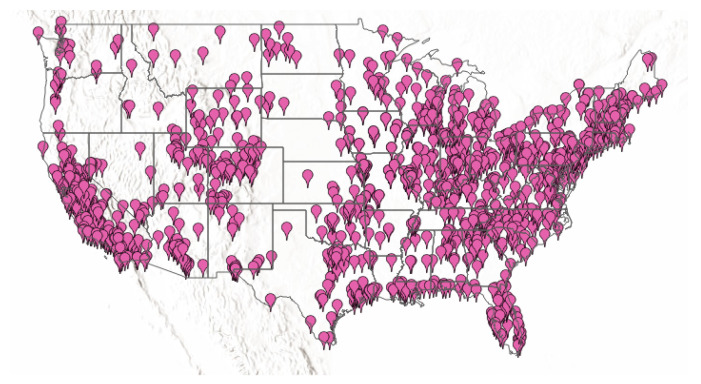
Positions of sensors, which collect information about SO${}_{2}$. Source: epa.gov/outdoor-air-quality-data/interactive-map-air-quality-monitors.

To assess our approach in this context, we chose an extensive real-world IoT database to analyze. This database is from the United States Environmental Protection Agency (US-EPA) (https://www.epa.gov/) (download available at aqs.epa.gov/aqsweb/airdata/download_files.html), which has millions of records (updated daily with new data) to four pollutants, Nitrogen Dioxide (NO${}_{2}$), Sulfur Dioxide (SO${}_{2}$), Carbon Monoxide (CO), and Ozone (O${}_{3}$). The database contains 28 fields described in Table 3. These data come from sensors around all US countries from the years 2000 until the present. We show in Figure 3 the position of the sensors in 2020, including information about SO${}_{2}$.

In this study, we use the Air Quality Index (AQI) as the observation variable. The AQI indicates how harmful the air is to human health. We show in Table 4 the AQI basics for ozone and particle pollution. In Table 4, the meaning of the colors is as follows: green, air quality is satisfactory, and air pollution poses little or no risk; yellow, air quality is acceptable, but there may be a risk for some people, particularly those who are unusually sensitive to air pollution; orange, members of vulnerable groups may experience health effects (the general public is less likely to be affected); red, some members of the general public may experience health effects, and members of sensitive groups may experience more serious health effects; purple, the risk of health effects is increased for everyone; maroon, health warning of emergency conditions, everyone is more likely to be affected.

The index for a pollutant is calculated using the mathematical expression of the Equation (5) [23].

(5) $$I_{P}=\frac{I_{Hi}-I_{LO}}{BP_{Hi}-BP_{LO}}\left(C_{P}-BP_{LO}\right)+I_{LO}$$

where,

$I_{P}$ is the index value for pollutant, *P*;
$C_{P}$ is the truncated concentration of pollutant, *P*;
$BP_{Hi}$ is the breakpoint that is ≥$C_{P}$;
$BP_{LO}$ is the breakpoint that is ≤CP;
$I_{Hi}$ is the AQI value corresponding to $BP_{Hi}$;
and, $I_{LO}$ is the AQI value corresponding to $BP_{LO}$.

In this context, we executed experiments aim to determine the areal units that optimize spatial autocorrelation patterns through the combined use of indicators of global spatial autocorrelation and the variance of local spatial autocorrelation. Furthermore, we applied the Kriging interpolation method for data visualization. Thus, we validate our approach, and at the same time, we contribute to solving a real-world problem.

### Study Areal Description

To evaluate the methods in these data, we chose two areal unit dimensions: a large one that involves the whole sensors described in Figure 3, and a small one, which includes the entire sensors in the state of California. We choose California due to the high variability between sensors’ values and the considerable number and distribution of sensors.

According to United Nations Statistics Division [52], the United States of America (USA) has a total area of 9,629,091 km${}^{2}$, and California is the third-largest by area at 423,970 km${}^{2}$ (it is also the most populous USA state). The surface in both areal unit dimensions were partitioned into hexagonal areal units, where each spatial unit aggregated the AQI’s pollutants. Furthermore, the hexagonal shape reduced the visual field bias when compared with the square units [53].

## 6. Computational Results

We implemented the experimental programs in Python (data prepossessing), and we made the geostatistic and spatial statistical methods in the R language; this made it possible to find all code and experimental data in our public repository (https://github.com/Leonild/SpatialDataAnalysis).

To evaluate our approach, first, we applied the framework described on Section 4.1 to determine the areal units that optimize the spatial autocorrelation patterns through the combined use of indicators of global and local spatial autocorrelation; this returns what the best areal unit to make data analysis is. Then, we applied the interpolation method described in Section 4.2, to an accurate data visualization. Furthermore, we compared the results with the works that use the classical statistics, to provide evidence that the analysis method could lead to wrong interpretations.

### 6.1. Spatial Statistics Analysis

Following Algorithm 1, we modeled the candidates’ areal units by regular hexagon shape, and we determined the length of the sides in five scales: 100 km, 200 km, 300 km, 400 km, and 500 km. Furthermore, we analyzed for all the pollutants, but here, due to the number of the images and very similar characteristics, we present results for only one pollutant (O${}_{3}$).

**Figure 4:**
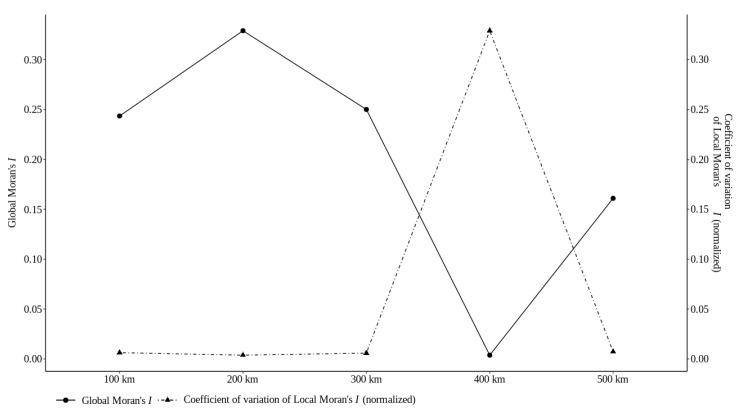
Trade-off between the global indicator of spatial association (Global Moran’s *I*) and the overall degree of structural (in)stability (coefficient of variation of Local Moran’s *I* normalized by scaling between the minimum and maximum values of the Global Moran’s *I* coefficients. Both global and local spatial statistics were computed for a row-standardized spatial weights matrix based on first-order rook contiguity.

Figure 4 shows Global Moran’s *I* coefficient and the coefficient of variation of Local Moran’s *I* for the areal units. Only some of the areal units show an improvement, with higher Global Moran’s *I* and lower coefficient of variation of Local Moran’s *I*. The other areal units just keep values that represent the absence of spatial autocorrelation and with high variation of Local Moran’s *I*. In this experiment, an areal unit of 200 km is linked to a higher pattern of spatial association and lower spatial heterogeneity than the other areal units; i.e., the former provides more consistent spatial patterns and is thus likely to reflect more reliable analytical results.

**Figure 5:**
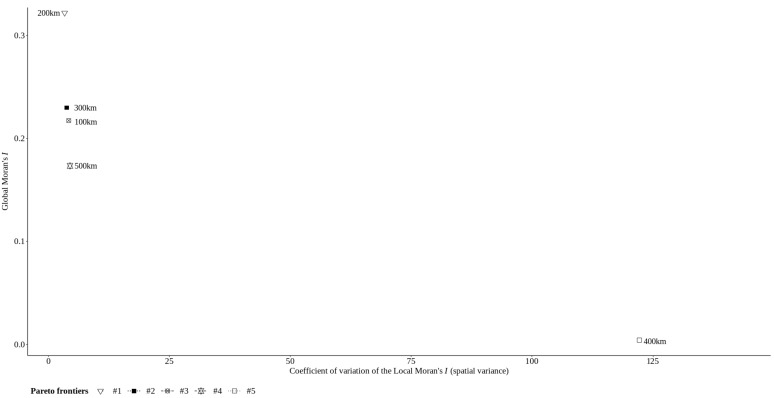
Pareto frontier and trade-off between Global Moran’s *I* and the coefficient of variation of Local Moran’s *I*.

To analyze the chart from Figure 5, we should remember the conflicting objectives that we considered; in this case, the ideal solution should have a higher Global Moran’s *I* (GM) and a lower coefficient of variation of Local Moran’s *I* (LM). Let us look at Figure 5. We have five possible areal units of data aggregated to choose for analyzing: (1) 100 km with a low LM and less high GM; (2) 300 km in the same context; (3) 500 km, which, however, has a low LM but also has a low GM; (4) the worst solution, 400 km, with a lower GM and a higher LM; and (5) the areal unit of 200 km with the higher GM and the lower LM. Therefore, according to the results of the multicriteria optimization framework in Figure 5, the Pareto-optimal solution is the areal units of 200 km. These areal units dominate the other ones because their criteria are better; i.e., they are combined with a higher Global Moran’s *I* and a lower coefficient of variation of Local Moran’s *I*. This means that the data aggregated inside the 200 km areal unit have a higher correlation than the others.

**Figure 6:**
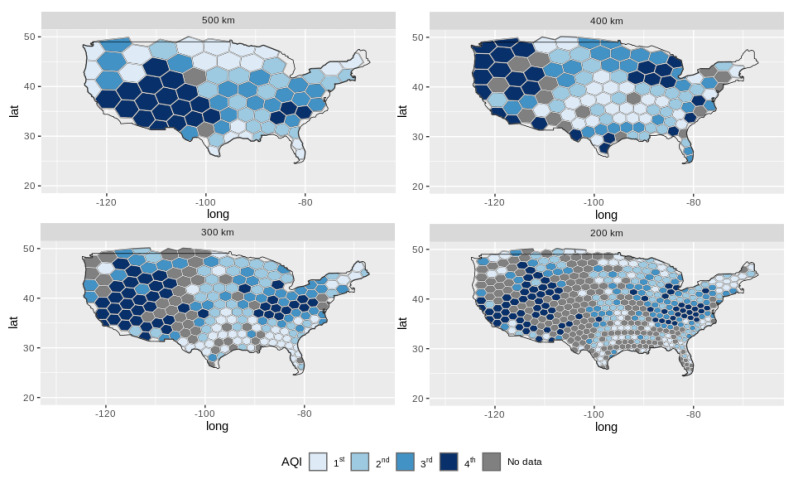
Comparison of spatial patterns of Pareto-optimal areal units with others arbitrary areal units. The patterns correspond to the ‘odds ratio measure’ of the frequency of geographic coordinates’ O${}_{3}$ data [54].

Figure 6 shows the spatial patterns of the O${}_{3}$ collected data from the geographic coordinates data sensors on the maps of the regular hexagons with the side lengths of 200 km, 300 km, 400 km, and 500 km. When we chose an arbitrary areal unit, such as 400 km or 500 km, we obtained different and discordant spatial patterns when compared with the Pareto-optimal areal units. In practice, this affects the conclusions and may lead to misunderstandings and mistakes by decision-makers when applying the strategy to the IoT infrastructure planning.

To analyze the method in another order of magnitude, we replicated the experiment to a smaller area, in which we used the same data but considered only the state of California. In this new experiment, we also modeled the candidates’ areal units by a regular hexagons shape; however, we determined the length of the sides in scales of 100 km, 90 km, 80 km, 70 km, 60 km, and 50 km.

**Figure 7:**
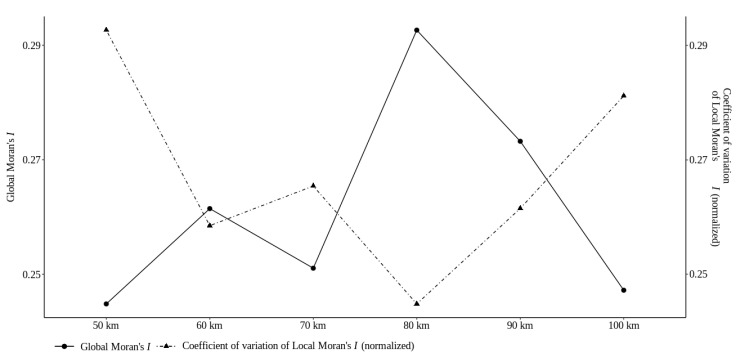
Trade-off between the global indicator of spatial association (Global Moran’s *I*) and the overall degree of structural (in)stability (coefficient of variation of Local Moran’s *I* normalized by scaling between the minimum and maximum values of the Global Moran’s *I* coefficients) considering the California states.

Figure 7 shows Global Moran’s *I* coefficient and the coefficient of variation of Local Moran’s *I* for the areal units in the California states. This makes it possible to observe that all the areal units show different patterns from each other. In this experiment, the areal unit of 80 km is linked to a higher pattern of spatial association and lower spatial heterogeneity than the other areal units; i.e., the former provides more consistent spatial patterns and is thus likely to reflect more reliable analytical results.

**Figure 8:**
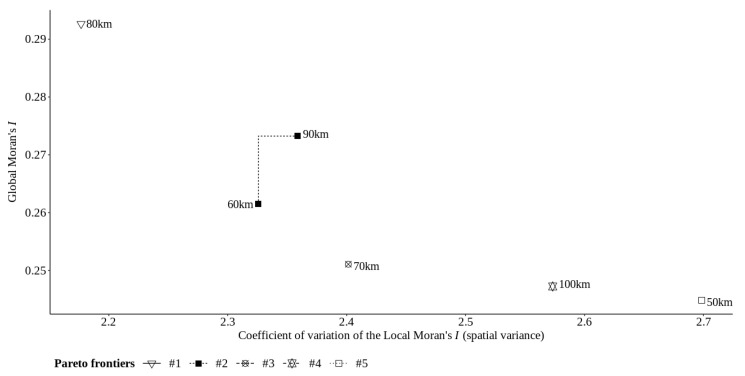
Pareto frontier and trade-off between Global Moran’s *I* and the coefficient of variation of Local Moran’s *I* for the O${}_{3}$ pollutant in California state.

To confirm the conclusion above, we present in Figure 8 the results of the multicriteria optimization framework, where the 80 km areal unit is alone in the first Pareto frontier. Moreover, it is also possible to observe that the 50 km areal unit is isolated in the last Pareto frontier; this means the lower pattern of spatial association and higher spatial heterogeneity than the other areal unit.

**Figure 9:**
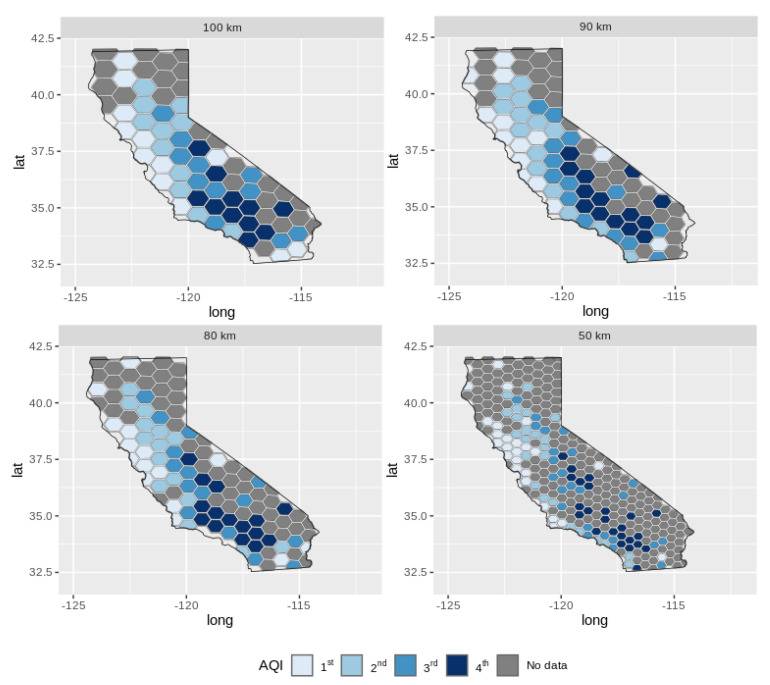
Comparison of spatial patterns of Pareto-optimal areal units with other arbitrary areal units in the state of California. The patterns correspond to the ‘odds ratio measure’ of the frequency of geographic coordinates O${}_{3}$ data [54].

Like Figure 9, Figure 8 shows the spatial patterns of the O${}_{3}$ collected data from the geographic coordinates data sensors on the maps of the regular hexagons with the side length of 100 km, 90 km, 80 km, and 50 km. If we chose an arbitrary areal unit, such as 50 km, we obtained different spatial patterns when compared with the Pareto-optimal areal units. It is essential to highlight that this affects the conclusions and may lead to misunderstandings and mistakes by decision-makers when applying the strategy to the IoT infrastructure planning.

### 6.2. Data Interpolation

To compare the results of the data interpolation with works that utilize classical statistics in the same context, we used data from 2015 related to O${}_{3}$ pollutants. Following the systematic method presented in Figure 2, first, we normalize the data, and then we build the fitted model. It is essential to remember that the map from the location is only for visualization.

**Figure 10:**
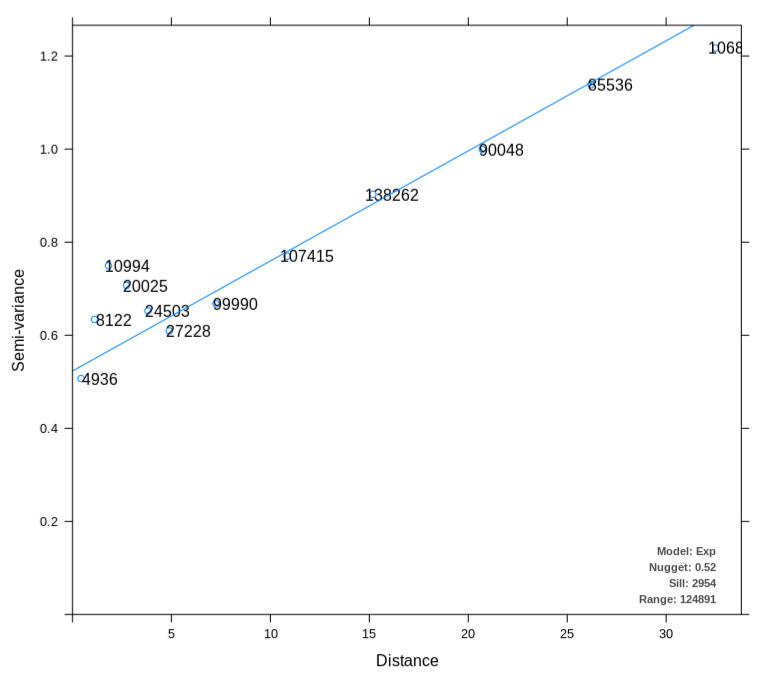
Variogram from the fitted model to O${}_{3}$ data in the United States in 2015.

We show in Figure 10 the fitted model used to apply the Kriging method. It can be observed that this variogram represents an exponential model; i.e., the spatial autocorrelation disappears entirely only at an infinite distance, which means that the near data are strongly autocorrelated.

**Figure 11:**
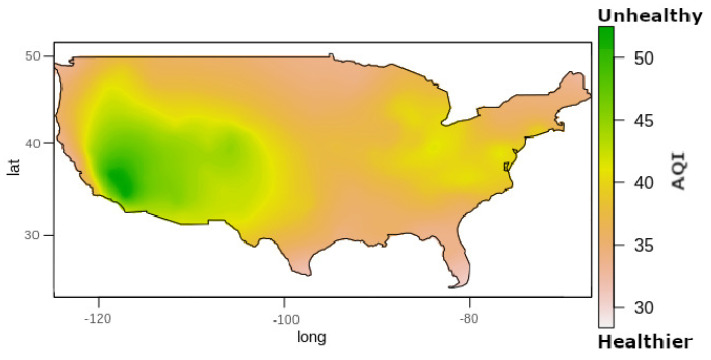
Kriging method interpolation applied to O${}_{3}$ AQI in the United States (2015).

**Figure 12:**
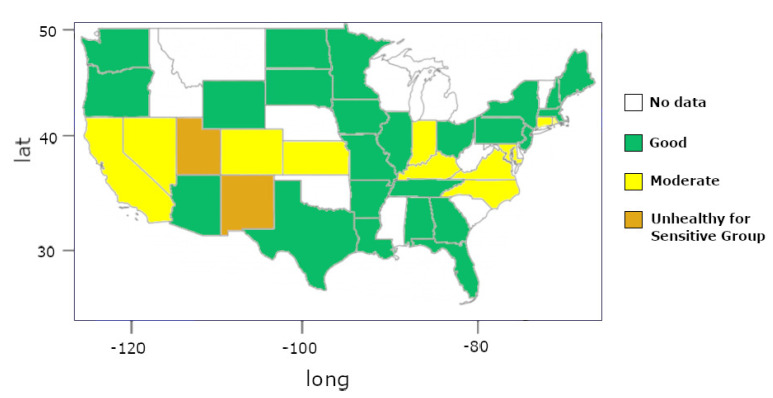
O${}_{3}$ AQI peer state in the United States in 2015 using classical statistics (average); the colors in the map follow the definitions in Table 4, and white means that the area does not have data information.

This fitted model is the input for Kriging interpolation. Figure 11 shows the result of Kriging interpolation to O${}_{3}$ data in the United States in 2015, where the gradient color represents the O${}_{3}$ AQI. If we chose an classical statistics methods to represent the same data (e.g., a simple average) like other literature works [23,24], we could obtain a map visualization like Figure 12; the colors in the map from Figure 12 follow the Table 4 definition.

It is possible to observe that if we consider only the mean by state (Figure 12), we can make incorrect interpretations about the data. For example, considering the average by country, we can conclude that entire state of California has air that could be a risk for some people, particularly those who are unusually sensitive to air pollution, which is not valid if we look to the interpolation data (Figure 10).

Another good example is the state of Arizona, which looks like a state with totally healthy air if we considered the map in Figure 12 (data collected in few points). However, we see in the interpolation map from Figure 11 that it is entirely incorrect to consider the Arizona state with entirely healthy air.

**Figure 13:**
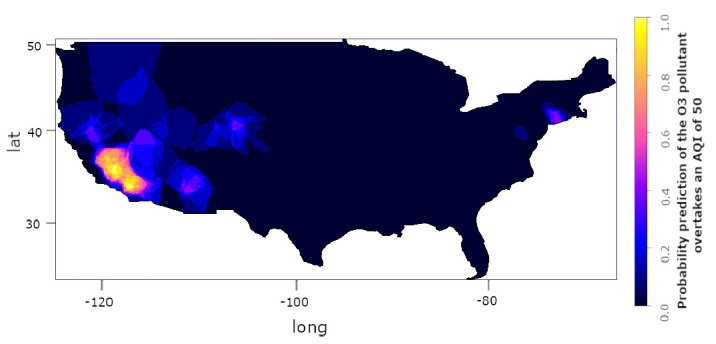
Kriging method indicative applied to O${}_{3}$ AQI in the United States (2015); the probability prediction that the O${}_{3}$ pollutant overtakes an AQI of 50.

With the geostatistics in our proposal (Kriging method), we can also estimate a prediction value; i.e., we can analyze the possibility of a factor that exceeds a predetermined amount. Figure 13 shows the probability prediction of the O${}_{3}$ pollutant overtaking an AQI of 50. The estimate floats from 0 (0%) to 1 (100%).

### 6.3. Discussion

By summarizing our results, we can observe that a classical statistical method is inadequate for data analysis of outdoor sensors. Furthermore, only a geostatistic or spatial static analysis may not be enough either. For this reason, we propose structuring several methods from geostatistics and spatial statistic aggregated with a multicriteria analysis to compose a systematic data analysis on outdoor sensors.

Although we present results only for the environmental context, our proposal is promising for a free contextual application in outdoor sensors’ data analysis. In the next section, we discuss our proposal’s limitation and future work.

## 7. Conclusions

The combination of devices with sensor networks and Internet access enables the communication between the physical world and cyberspace, providing the development of solutions to many real-world problems through the IoT.

IoT involves anything with network access with or without human interaction required, and the data from these “things” can be provided in many forms, such as streaming and discrete data, images, and social media, among others. The combination of the network of sensors with the Internet enables the communication between the virtual and real world, allowing the decision making without human intervention. However, a wrong decision due to poor data quality or erroneous data interpretation can cause significant financial harm to companies and institutions.

The problem of data quality becomes complex and controversial with the evolution of technology. The data quality and data accuracy are also related to the data analysis [7,8,9]. In this context, we presented in this paper a systematic approach to support the data analysis by considering the sensor spatiality factor and geographic aspects. Moreover, we applied the methods on an extensive real-world database from the United States Environmental Protection Agency (US EPA).

First, we determined the areal units that optimize the spatial autocorrelation patterns through the combined use of indicators of global and local spatial autocorrelation, which showed what the best areal unit to make data analysis is. Next, we applied the Kriging interpolation to an accurate data visualization, and we also provided evidence that the report given only by the classical statistics could lead to wrong interpretations.

Although we validate our proposed method only in the environmental context, we could apply this analysis in any context, including a free-context method. However, to validate it as it would be validated with a free-context method, we would need to realize these specific analyses. Furthermore, it is important to highlight some limitations in the experiments:

- We only did offline experiments.
- Due to the analysis time, we could not use this method in critical applications without substantial modifications.
- It is necessary to validate this method in other contexts to ensure that our proposals have a free context application.

In future work, we intend to perform experiments and analysis in micro-regions with other study cases, where we hope to evaluate the decision-making as well. Furthermore, we also aimed to apply the spatial autocorrelation to deduce the correct spatial distributed sensor dimensions. In another context, we intend to do a performance evaluation to conclude if it is feasible to use our approach in real-time execution for critical applications.

## Figures and Tables

**Table 1:** Main features of the related works.

Related Work	Environment	QoD	Multi-Criteria Analysis	Spatial
**Antonic, A. et al. [10]**	Simulator	X	X	X
**Alam, S. and Noll, J. A. [11]**	Simulator	X	X	X
**Kothari, A. et al. [12]**	Simulator	*√*	X	X
**Karkouch, A. et al. [13]**	Simulator	X	X	X
**Xu, X.; Lei, Y.; and Li, Z. [17]**	Real World	*√*	X	X
**Cheng, H. et al. [18]**	Simulator	*√*	X	X
**Liu, Q. [22]**	Real World	X	X	X
**Li, Z. et al. [24]**	Real World	X	X	X
**Habibia, R. [37]**	Simulator	X	X	*√*
**de Andrade, S.C. et al. [38]**	Real World	X	*√*	*√*
**This paper**	Real world	*√*	*√*	*√*

**Table 2:** List of important notation.

Term	Description
$w_{ij}$	matrix unit weight
$y_{i}$	the value of interest on location
$\bar{y}$	the mean of interest on location
*n*	the total observations
*I*	the Moran’s index
$I_{i}$	the Moran’s LISA for each map unit
*X*	a set of any areal units with different levels of data aggregation
ϕ	objective functions
$Z(S_{i})$	a known value at the location
$\lambda_{i}$	an unknown weight for the measured value at the location
$S_{0}$	the location with data unknown to prediction
*N*	the number of measured values

**Table 3:** Description of the EPA database 28 fields.

Database Fields			
1	Index	15	O${}_{3}$ Unit
2	State Code	16	O${}_{3}$ 1st Max Value
3	County Code	17	O${}_{3}$ 1st Max Hourn
4	Site Num (Local in a county)	18	O${}_{3}$ AQI
5	Adress (Street, number…)	19	SO${}_{2}$ Units (description)
6	State (name)	20	SO${}_{2}$ Mean
7	County (name)	21	SO${}_{2}$ 1st Max Value
8	City (name)	22	SO${}_{2}$ 1st Max Hourn
9	Date Local	23	SO${}_{2}$ AQI
10	NO${}_{2}$ Units (description)	24	CO Units (description)
11	NO${}_{2}$ Mean	25	CO Mean
12	NO${}_{2}$ 1st Max Value	26	CO 1st Max Value
13	NO${}_{2}$ 1st Max Hourn	27	CO 1st Max Hourn
14	NO${}_{2}$ AQI	28	CO AQI

**Table 4:** AQI basics for Ozone and Particle Pollution. Source: www.airnow.gov/aqi/aqi-basics.

AQI Color	Levels of Concern	Values of Index
Green	Good	0 to 50
Yellow	Moderate	51 to 100
Orange	Unhealthy for Sensitive Groups	101 to 150
Red	Unhealthy	151 to 200
Purple	Very Unhealthy	201 to 300
Maroon	Hazardous	301 and higher

## Data Availability

Publicly available datasets were analyzed in this study. This data can be found here: aqs.epa.gov/aqsweb/airdata/download_files.html.
